# Neurotoxicity in Preclinical Models of Occupational Exposure to Organophosphorus Compounds

**DOI:** 10.3389/fnins.2016.00590

**Published:** 2017-01-18

**Authors:** Jaymie R. Voorhees, Diane S. Rohlman, Pamela J. Lein, Andrew A. Pieper

**Affiliations:** ^1^Department of Psychiatry, University of Iowa Carver College of MedicineIowa City, IA, USA; ^2^Interdisciplinary Graduate Program in Human Toxicology, University of Iowa Carver College of MedicineIowa City, IA, USA; ^3^Department of Occupational and Environmental Health, University of Iowa College of Public HealthIowa City, IA, USA; ^4^Department of Molecular Biosciences, School of Veterinary Medicine, University of California, DavisDavis, CA, USA; ^5^Department of Neurology, University of Iowa Carver College of MedicineIowa City, IA, USA; ^6^Department of Free Radical and Radiation Biology Program, University of Iowa Carver College of MedicineIowa City, IA, USA; ^7^Department of Radiation Oncology Holden Comprehensive Cancer Center, University of Iowa Carver College of MedicineIowa City, IA, USA; ^8^Department of Veteran Affairs, University of Iowa Carver College of MedicineIowa City, IA, USA; ^9^Weill Cornell Autism Research Program, Weill Cornell Medical CollegeNew York, NY, USA

**Keywords:** neurotoxicity, organophosphorus compounds, organophosphate pesticides, organophosphates, organophosphate ester pesticides

## Abstract

Organophosphorus (OPs) compounds are widely used as insecticides, plasticizers, and fuel additives. These compounds potently inhibit acetylcholinesterase (AChE), the enzyme that inactivates acetylcholine at neuronal synapses, and acute exposure to high OP levels can cause cholinergic crisis in humans and animals. Evidence further suggests that repeated exposure to lower OP levels insufficient to cause cholinergic crisis, frequently encountered in the occupational setting, also pose serious risks to people. For example, multiple epidemiological studies have identified associations between occupational OP exposure and neurodegenerative disease, psychiatric illness, and sensorimotor deficits. Rigorous scientific investigation of the basic science mechanisms underlying these epidemiological findings requires valid preclinical models in which tightly-regulated exposure paradigms can be correlated with neurotoxicity. Here, we review the experimental models of occupational OP exposure currently used in the field. We found that animal studies simulating occupational OP exposures do indeed show evidence of neurotoxicity, and that utilization of these models is helping illuminate the mechanisms underlying OP-induced neurological sequelae. Still, further work is necessary to evaluate exposure levels, protection methods, and treatment strategies, which taken together could serve to modify guidelines for improving workplace conditions globally.

## Introduction

Organophosphorus chemicals (OPs) were developed in the early 1900s as insecticides that disabled insects via inhibition of cholinesterases (ChE). While these chemicals were equally as effective as their predecessor organochlorine pesticides, OPs offered a significant advantage of reduced environmental persistence (Marrs et al., 2007; Moshiri et al., 2012). Unfortunately, because of the conservation of ChEs across species, including human, OPs were adapted as chemical warfare agents in the 1930s and remain among the most potent chemical warfare agents used today. Moreover, intentional ingestion of OP pesticides is the most common means of suicide in regions of the world where guns are not widely available (Eddleston et al., 2008). Throughout the world, however, OPs have primarily been used as insecticides to protect crops, animals, and humans, and also as industrial solvents in manufacturing or as fuel additives (Davisson et al., 2005). For more general information on OPs see: (Pope, 1999; Costa, 2006; Balali-Mood and Abdollahi, 2014). Because of their widespread use, OPs are ubiquitous in the global environment, as evidenced by their detection in rivers, groundwater, soil, air, plants, animals, and human tissues (Schnoor, 1992; Davisson et al., 2005; Barr et al., 2011; Clune et al., 2012). Although ambient exposure to OPs in some parts of the world has declined due to recent restrictions (Clune et al., 2012), occupational OP exposures are still prevalent globally, and neurotoxicity has emerged as one of the primary endpoints of concern (Rohlman et al., 2011). Continued research is needed to address the neurotoxicity associated with occupational OP exposures, in order to determine safe exposure levels, appropriate protection methods, and promising treatment strategies. Here, we review *in vivo* studies that have modeled occupational OP exposure to determine the impact on the nervous system, as well as *in vitro* studies designed to examine molecular pathways relevant to occupational OP exposure.

The multiple clinical syndromes associated with OPs are largely determined by the dose, route, and duration of OP exposure. In this review, we distinguish between the following types of OP exposure: acute (single exposure or multiple exposures within 24 h), developmental (*in utero* or early life exposures*)*, prolonged/subchronic (exposures of <90 days), and chronic (>90 days) (Klaassen et al., 2013). Acute OP exposures that inhibit AChE enzyme activity by 80–90% precipitate cholinergic crisis via overstimulation of the nervous system leading to respiratory failure, flaccid paralysis, decreased blood pressure, parasympathetic discharge, and even death. For reviews on acute OP exposure (see Sullivan and Blose, 1992; Singh and Sharma, 2000; Peter et al., 2014). The effects of developmental, prolonged, and chronic exposures to OPs are less clear, however. One challenge in studying these types of exposures is their proper characterization. Currently, AChE activity and personal reports are the most commonly used methods for assessing OP exposures. However, baseline AChE activity levels vary greatly, reductions in AChE activity do not correlate well with reported symptoms or observed deficits, manifestations of exposure persist long after ChE levels return to normal, and personal accounts of exposure may be incomplete (Rohlman et al., 2011, 2016). Fortunately, studies are currently underway to specifically expand the repertoire of biomarkers for OP exposure (Lein et al., 2012).

Prolonged or chronic, low- to moderate-level exposures frequently occur in occupational settings, but also include living with someone who is occupationally exposed, ingesting contaminated food, or living near OP application and manufacturing sites. Numerous occupations result in direct or indirect OP exposure, including agricultural work and pesticide manufacturing. Worldwide, billions of pounds of pesticides are manufactured, transported, and applied yearly, and individuals working at any stage of pesticide production, transportation, or application are at risk for OP exposure. Other individuals at high risk of occupational OP exposure include exterminators, greenhouse workers and florists, aircraft personnel exposed to jet fuel and engine oil, veterinarians exposed while treating animals for pests, and military personnel exposed during deployment. Each of these occupations represents a different profile of occupational OP exposure, with the potential for different neurotoxic outcomes. For reviews concerning low-level, chronic exposures to OP or chronic AChE inhibition, (see Steenland, 1996; Brown and Brix, 1998; Ray, 1998; Ray and Richards, 2001; Kamel and Hoppin, 2004). Chronic exposures vary widely in duration, route, dose, and severity of effects. Unfortunately, studies regarding the effects of these types of exposures in humans are controversial. Though chronic exposure does not usually induce symptoms associated with acute cholinergic overstimulation, it is associated with debilitating neuropsychiatric conditions, such as depression, anxiety, and suicide (Stephens et al., 1995; Parrón et al., 1996; Salvi et al., 2003; Stephens and Sreenivasan, 2004; Lee et al., 2007; Beseler et al., 2008; Freire and Koifman, 2013; Zaganas et al., 2013; Beard et al., 2014). Furthermore, occupational exposures are associated with deficits in executive functioning (Fiedler et al., 1997; Baldi et al., 2003; Farahat et al., 2003; Rohlman et al., 2007; Ross et al., 2013; Zaganas et al., 2013), increased prevalence of neurodegenerative diseases, such as Parkinson's disease (PD), Alzheimer's disease (AD), and amyotrophic lateral sclerosis (ALS) (Mcdowell et al., 1994; Priyadarshi et al., 2001; Baldi et al., 2003; Alavanja et al., 2004; Santibáñez et al., 2007; Kanthasamy et al., 2012; Malek et al., 2012; Narayan et al., 2013; Wang et al., 2014), psychiatric conditions, such as depression, anxiety, and suicide (Stephens et al., 1995; Parrón et al., 1996; Salvi et al., 2003; Stephens and Sreenivasan, 2004; Lee et al., 2007; Beseler et al., 2008; Freire and Koifman, 2013; Zaganas et al., 2013; Beard et al., 2014; Hardos et al., 2016), and cognitive deficits (Fiedler et al., 1997; Baldi et al., 2003; Farahat et al., 2003; Rohlman et al., 2007; Ross et al., 2013). Conversely, some studies have reported no strong associations between occupational exposures and neurobehavioral performance (Starks et al., 2012) or neurodegeneration (Baltazar et al., 2014; Sánchez-Santed et al., 2016). Furthermore, a recent study observed no correlation between urinary OP metabolites and neurobehavioral performance (Krieg, 2013). Interpretation of the epidemiological findings is complicated by the complexity of chemical exposures, poor exposure records, problems with bias and recall in self-reporting, and the fact that some forms of exposure result in delayed neuropathies that may not manifest within the timeline of the study. Thus, controlled *in vivo* and *in vitro* studies in preclinical models are important for establishing cause-effect relationships between OP exposures that simulate occupational exposure and adverse outcomes relevant to people.

In this review, “human acute exposure” is defined as a single accidental exposure or multiple exposures within 24 h. “Human subchronic exposure” is defined as repeated exposures over weeks, and “human chronic exposure” is defined as repeated exposures over months or years. The same criteria for duration of exposure was applied to the *in vivo* animal studies examined here. To assess the current body of literature reporting effects of prolonged and chronic OP exposures in preclinical models, we conducted a comprehensive literature search of the PubMed electronic bibliographic database system of the National Library of Medicine of the United States using the following terms without date restrictions: (1) duration of exposure (chronic, continuous, repeated, prolonged, multiple, or extended); (2) dose (subthreshold, subcholinergic, subclinical, low, moderate, or occupational) and route (dermal, topical, subcutaneous, or inhalation); and (3) OP and the names of specific OPs (see Table 1). We found that chlorpyrifos (CPF), diazinon (DZ), dichlorvos (DDVP), diisopropylfluorophosphate (DFP), malathion, methylparathion (MP), and triorthocresyl phosphate (T_*O*_CP) were the most extensively studied OPs, and that many studies identified a specific OP as a key search term without referencing the general class of chemicals. Studies included in this review and deemed as low to moderate exposures were prolonged, chronic, or repeated exposures that did not cause cholinergic crisis or overt signs of toxicity, including significant changes in weight or ChE inhibition >85%. We focused on dermal exposure, as this is the dominant route of exposure in occupational settings (Durham et al., 1972; Sullivan and Krieger, 1992; Fenske et al., 2012). The rate of dermal penetration and absorption varies greatly between OP agents, and the toxicokinetics of OPs absorbed through the skin can be different from those that occur by other routes of exposure. Intraperitoneal (IP) injection and oral administration, for example, achieve higher systemic dosing than dermal exposure (Ellison et al., 2011). Although inhalation is also a significant route of occupational exposure, especially when considering aircraft crew and maintenance workers exposed to T_*O*_CP (de Ree et al., 2014; Hardos et al., 2016), we found a limited number of published OP inhalation animal studies. Given that it is difficult to draw meaningful conclusions from so few studies, we did not review the inhalation route of exposure. Furthermore, *in vivo* studies were excluded if exposures occurred during development, prior to 5 weeks of age for mice (Finlay and Darlington, 1995) and rats (Sengupta, 2013). Finally, we used the percent inhibition of AChE or ChE as a dosimetric for comparing studies, as many of these studies indicate blood, cell, or brain region-specific levels of ChE inhibition. Table 2 (*in vivo* studies) and Table 3 (*in vitro* studies) list the studies included in this review.

**Table 1:** **Commonly used and studied organophosphorous chemicals**.

**Structure**	**Uses**	**Trade names**	**CAS number**	**Class**	***In vivo*** **studies**	***In vitro/Ex vivo*** **studies**
Chlorpyrifos (C_9_H_11_Cl_3_NO_3_PS) 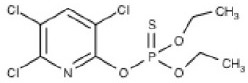	Agricultural (cotton, corn, grain, and fruit trees), residential, and Commercial insecticide	Brodan, Chlorpyrifos-ethyl, Detmol UA, Dowco 179, Dursban, Empire, Eradex, Lorsban, Paqeant, Piridane, Scout, Stipend, and Tricel	2921-88-2	Thiophosphoric acid	Huff et al., 2001; Terry et al., 2003, 2007; Mitra et al., 2009; Middlemore-Risher et al., 2010; Chen et al., 2011, 2014; Lim et al., 2011; Speed et al., 2012; Terry, 2012; Muller et al., 2014; Hernandez et al., 2015; Lee et al., 2016	Howard et al., 2005; Parran et al., 2005; Mense et al., 2006; Gearhart et al., 2007; Prendergast et al., 2007; Yang et al., 2008; Grigoryan and Lockridge, 2009; Rush et al., 2010; Slotkin and Seidler, 2010; Middlemore-Risher et al., 2011
Diazinon (C_12_H_21_N_2_O_3_PS) 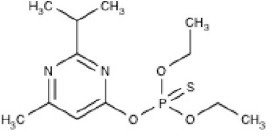	Agricultural (rice, corn, tobacco, and potatoes), residential, and commercial insecticide	Basudin, Dazzel, Gardentox, Kayazol, Knox Out, Nucidol, and Spectracide	333-41-5	Thiophosphoric acid		Rush et al., 2010; Slotkin and Seidler, 2010; Pizzurro et al., 2014a,b; Colovic et al., 2015
Dichlorvos (C_4_H_7_Cl_2_O_4_P) 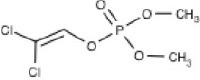	Agricultural, residential, and commercial insecticide	Apavap, Benfos, Cypona, DDVP, Devikol, Didivane, Duarvos, Ekastrel, Marvex, Prentox, Vapona, Verdipor, and Verdisol	62-73-7	Phosphoric acid	Kaur et al., 2007; Verma et al., 2009; Binukumar et al., 2010, 2011	
Diisopropylfluorophosphate (C_6_H_14_FO_3_P) 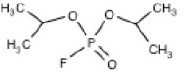	Miotic medicine, nerve agent, and insecticide	Difluorophate, Diflupyl, Diflurphate, Dyflos, Dyphlos, Fluropryl, Fluostigmine, isofluorophate, Neoglaucit, PF-3, and T-1703	55-91-4	Diisopropylfluoro-phosphoric acid	Bushnell et al., 1991; Mundy et al., 1993; Prendergast et al., 1997, 1998; Terry et al., 2011, 2014; Terry, 2012	Gearhart et al., 2007; Gao et al., 2016
Malathion (C_10_H_19_O_6_PS) 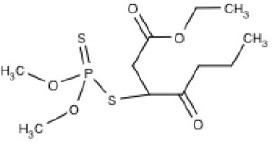	Medical (head and body lice), Agriculture, residential, and commercial insecticide	Cythion, Fyfanon	121-75-5	Dithiophosphoric acid		Balbuena et al., 2010, 2011
Methyl parathion (C_10_H_14_NO_5_PS) 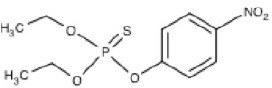	Nerve agent and agricultural insecticide (cotton, rice, and fruit trees)	Bladan M, Dalf, Dimethyl Parathion, E 601, Gearphos, Kilex Parathion, ME605, Metaphos, Nitrox 80, Partron M, and Penncap-M	298-00-0	Thiophosphoric acid	Castillo et al., 2002; Ma et al., 2003; Sun et al., 2006	Rocha et al., 1996; Yousefpour et al., 2006; Berríos et al., 2015
Tri-orthy-cresyl-phosphate (C_21_H_21_O_4_P) 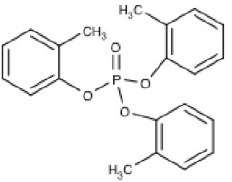	Plasticizer	TCP, ToCP, Tri-o-cresyl ester of phosphoric acid, Tri-o-cresyl phosphate	78-30-8	Tri-ortho-cresyl-phosphoric acid		Chen et al., 2013; Hausherr et al., 2014; Duarte et al., 2016; Hausherr et al., 2016

**Table 2:** *****In vivo*** studies included in this review**.

**Organophosphorous chemical**	**Dosing parameters**		**% ChE reductions**	**Species**	**Publication**
	**Dose (mg/kg)**	**Exposure period**			
Chlorpyrifos (CPF)	0.1, 1, 10	7 days daily	0–80 in plasma	Wistar rats	Muller et al., 2014
	2.5, 10, 18	30 days every other day	60–80 in plasma	Wistar rats	Terry et al., 2007
	2.5, 5, 10, 20	10 days daily	N/A	Sprague-Dawley rats	Chen et al., 2014
	2.5, 10, 18, 25	1–38 days daily	30–60 in plasma	Wistar rats	Terry et al., 2003
	2.5, 30	Once a week for 4 weeks	0–60 in plasma	Sprague-Dawley rats	Huff et al., 2001
	3, 10	21 days daily	90–100 in whole blood	Long Evans rats	Lee et al., 2016
	3, 18	14 days daily	60–80 in brain	Wistar rats	Hernandez et al., 2015
	5	5 days daily	40 in brain	C57B1/6J mice	Speed et al., 2012
	10, 18	30 days every other day	80 in plasma	Wistar rats	Terry, 2012
	10, 20, 40	7 days daily	N/A	Sprague-Dawley rats	Chen et al., 2011
	18	14 days daily	70 in plasma	Wistar rats	Middlemore-Risher et al., 2010
	18	30 days every other day	80 in plasma	Wistar rats	Middlemore-Risher et al., 2010
	20.2, 40.4	7 days daily	30 in serum	Swiss albino mice	Lim et al., 2011^*^
	40.4, 101	18 days daily	75–95 in serum	Swiss albino mice	Mitra et al., 2009^*^
Dichlorvos (DDVP)	1, 6	84 days daily	10–55 in serum	Wistar rats	Verma et al., 2009
	2.5	84 days daily	0 in brain	Wistar rats	Binukumar et al., 2010
	2.5	84 days daily	N/A	Wistar rats	Binukumar et al., 2011
	6	84 days daily	N/A	Wistar rats	Kaur et al., 2007
Diisopropylfluorophosphate (DFP)	0.05, 0.25, 0.5	14 days daily	50 in brain	Wistar rats	Prendergast et al., 1997
	0.1, 0.2	21 days daily	50–75 in brain	Long Evans rats	Bushnell et al., 1991
	0.2, 0.4	5 days a week for 4 weeks	70–85 in brain	Long Evans rats	Mundy et al., 1993
	0.25	14 days daily	N/A	Wistar rats	Prendergast et al., 1998
	0.25	14 days daily	50–60 in brain	Wistar rats	Stone et al., 2000
	0.25, 0.5, 0.75, 1.0	30 days every other day	55–80 in plasma	Wistar rats	Terry et al., 2011
	0.25, 0.75	30 days every other day	75 in plasma	Wistar rats	Terry, 2012
	0.5	30 days every other day	40 in plasma	Wistar rats	Terry et al., 2014
	0.8	3 days a week for 4 weeks	70–85 in brain	Long Evans rats	Mundy et al., 1993
Malathion	30, 100	15 days daily	0–40 in brain	Swiss albino mice	dos Santos et al., 2016
Methyl Parathion (MP)	0.1, 1	95 days daily	3–64 in brain	Sprague-Dawley rats	Ma et al., 2003^*^^#^
	2	10 days daily	30 in plasma	Wistar rats	Castillo et al., 2002
	3	21 days daily	80–90 in brain	Sprague-Dawley rats	Sun et al., 2003
	3	21 days daily	80 in brain	Sprague-Dawley rats	Sun et al., 2006

* Indicates route of exposure other than SC (topical or tail patch);

# *Indicates test subjects were female*.

**Table 3:** *****In vitro*** studies included in this review**.

**OP**	*****In vitro/Ex vivo*** model system**	**Exposure parameters**		**% ChE reductions**	**Publication**
		**Concentration (μM)**	**Exposure period**		
Chlorpyrifos (CPF)	Primary Culture (superior cervical ganglia)	0.0001, 0.001, 0.01, 0.1, 1, 10	24 h	≤90	Howard et al., 2005
	Primary Culture (dorsal root ganglia)	0.01, 0.1, 1, 10 (CPF); 0.01, 0.1, 1, 10 (CPO)	24 h	≤75	Yang et al., 2008
	BMEC; Primary Culture (astrocytes)	0.01, 0.1, 1, 10, 100, 1000, 10,000	24 h	40–100	Parran et al., 2005
	PC12 (rat adrenal pheochromocytoma cell line)	0.1, 1, 10, 50 (CPF, CPO)	20 min	0 (CPF); ≥50 (CPO)	Meijer et al., 2014
	PC12 (rat adrenal pheochromocytoma cell line)	0.1–100	24 h	N/A	Meijer et al., 2015
	Hippocampal Slice Cultures	0.1, 1, 10 (CPO)	1, 3, or 7 days	≤50	Prendergast et al., 2007
	Primary Culture (cortical neurons)	1, 5, 10, 20 (CPF, CPO)	1 or 24 h	0–50	(Middlemore-Risher et al., 2011)
	Primary Culture (cortical neurons)	100 (CPF); 1, 10, 30, 100 (CPO)	24 h	80–90	Rush et al., 2010
	PC12 (rat adrenal pheochromocytoma cell line)	30	1–3 days	N/A	Slotkin and Seidler, 2010
	Isolated kinesin and microtubules	1, 10	20 min	N/A	Gearhart et al., 2007
	Isolated tubulin and microtubules	5, 10, 25, 50, 100	24 h	N/A	Grigoryan and Lockridge, 2009
	Human fetal primary astrocytes	0, 25, 50, 100	14 days, 7 days apart	N/A	Mense et al., 2006
Diazinon (DZ)	Isolated synaptosomes	0.00000001-0.001 (DZ, DZO, IMP)	1 h	≤95	Colovic et al., 2015
	Primary Culture (cortical astrocytes/hippocampal neurons)	0.1, 1, 10 (DZ,DZO)	24 h	50–80	Pizzurro et al., 2014a
	Primary Culture (cortical astrocytes/hippocampal neurons)	0.1, 1, 10 (DZ,DZO)	24 h	20 in astrocytes	Pizzurro et al., 2014b
	Primary Culture (cortical neurons)	1, 10, 30, 100 (DZ, DZO)	24 h	≤90	Rush et al., 2010
	PC12 (rat adrenal pheochromocytoma cell line)	30	1–3 days	N/A	Slotkin and Seidler, 2010
Diisopropylfluorophosphate (DFP)	Primary Culture (cerebral cortices)	0.0001, 0.001, 0.01, 0.1, 1	24 h	N/A	Gao et al., 2016
	Isolated kinesin and microtubules	0.0001, 0.0059	20 min	N/A	Gearhart et al., 2007
Malathion	RBE4 cells	0.00001 (malathion); 0.000001 (malaoxon)	2, 4, 8, 16, and 26 h	N/A	Balbuena et al., 2011
	RBE4 cells; BMEC	0.01, 0.1, 1, 10, 100, 1000	24 h	30–80	Balbuena et al., 2010
Parathion	Primary Culture (hippocampal neurons)	30, 40, 50, 100 (POX)	single dose for 3 weeks	N/A	Yousefpour et al., 2006
	Primary Culture (hippocampal neurons)	0.3, 3, 30, 300 (POX)	≤1 h	N/A	Rocha et al., 1996
	PC12 (rat adrenal pheochromocytoma cell line)	3, 10, 50	20 min	≤50	Meijer et al., 2014
	Primary Culture (differentiating neural and stem progenitors)	16, 30, 50, 66, 100, 150, 200, 300 (POX)	6 days	≤50	Berríos et al., 2015
Tri-ortho-cresyl phosphate (T*o*CP)	Primary Culture (corticol neurons)	0.001, 0.01, 0.1, 1, 10	24 h	N/A	Hausherr et al., 2014
	Primary Culture (corticol neurons)	10	24 h	N/A	Hausherr et al., 2016
	Primary Culture (corticol neurons)	10	48 h	0	Duarte et al., 2016
	SH-SY5Y (human derived neuroblastoma cell line)	200, 500, 1000	24 h	N/A	Chen et al., 2013

*BMEC, bovine brain microvascular endothelial cell line; RBE4, rat brain microvascular endothelial cell line*.

## Neurobehavioral effects in laboratory models

Hereafter, we review the neurobehavioral findings of studies aimed at understanding prolonged or repeated, low- to moderate-level OP exposure in animal models. We examine OP effects on learning and memory, attention, impulsivity, motility, depression, and anxiety. See Table 2 for a summary of the studies included, and Table 4 for a brief summary of neurobehavioral findings.

**Table 4:** **Summary of behavioral studies included in this review**.

**Behavioral domain**	**OP**	**Behavioral outcome**	**Species**	**Publication**
Learning and memory	CPF	Deficit (MWM)	Wistar rats	Terry et al., 2003
		Deficit (MWM)	Wistar rats	Terry et al., 2007
		Deficit (MWM and RAM)	Wistar rats	Terry, 2012
	DDVP	Deficit (MWM and OC passive avoidance)	Wistar rats	Verma et al., 2009
	DFP	Deficit (OC)	Long Evans rats	Bushnell et al., 1991
		Deficit (MWM)	Wistar rats	Prendergast et al., 1997
		Deficit (MWM); No Deficit (recall)	Wistar rats	Prendergast et al., 1998
		Deficit (MWM)	Wistar rats	Stone et al., 2000
		Deficit (MWM and NOR)	Wistar rats	Terry et al., 2011
		Deficit (MWM); No Deficit (RAM)	Wistar rats	Terry, 2012
	MP	Deficit (MWM)	Wistar rats	Castillo et al., 2002
		No Deficit (OC active avoidance)	Sprague-Dawley rats	Sun et al., 2006
	Malathion	Deficit (NOR)	Swiss albino mice	dos Santos et al., 2016
Attention and impulsivity	CPF	Deficit (5C-SRTT)	Wistar rats	Middlemore-Risher et al., 2010
	DFP	Deficit (5C-SRTT)	Wistar rats	Terry et al., 2014
Motility and motor coordination	CPF	Deficit (grip strength)	Wistar rats	Terry et al., 2003
		No Deficit (activity)	Sprague-Dawley rats	Chen et al., 2014
	DDVP	Deficit (grip strength and motor coordination)	Wistar rats	Verma et al., 2009
		Deficit (activity)	Wistar rats	Binukumar et al., 2010
	DFP	Deficit (activity)	Long Evans rats	Bushnell et al., 1991
		Deficit (activity)	Wistar rats	Prendergast et al., 1997
		Deficit (grip strength); No Deficit (motor coordination)	Wistar rats	Terry et al., 2011
	MP	Deficit (activity and motor coordination)	Sprague-Dawley rats	Sun et al., 2006
Depression and anxiety	CPF	Deficit (exploration)	Wistar rats	Terry et al., 2003
		Deficit (exploration)	Wistar rats	Terry et al., 2007
		Deficit (FST, EPM and novelty-suppressed feeding)	Sprague-Dawley rats	Chen et al., 2011
		Deficit (FST and learned helplessness)	Sprague-Dawley rats	Chen et al., 2014
	DDVP	Deficit (exploration)	Wistar rats	Binukumar et al., 2010
	DFP	Deficit (exploration)	Wistar rats	Prendergast et al., 1998
		Deficit (exploration)	Wistar rats	Stone et al., 2000
		Deficit (exploration)	Wistar rats	Terry, 2012

*5C-SRTT, 5 choice- serial reaction time test; CPF, chlorpyrifos; DDVP, dichlorvos; DFP, diisopropyl-fluorophosphate; EPM, elevated-plus maze; FST, forced swim task; MP, methyl parathion; MWM, Morris water maze; NOR, novel object recognition; OC, operant conditioning; RAM, radial arm maze*.

### Learning and memory

Epidemiological studies have linked occupational OP exposure to deficits in both learning and memory, including self-reported memory and standardized tasks of information processing (Farahat et al., 2003; Rohlman et al., 2007; Ismail et al., 2012). Conversely, some studies have found no association between occupational OP exposure and learning and memory deficits (Baltazar et al., 2014; Sánchez-Santed et al., 2016). Accordingly, researchers have investigated this phenomenon in various *in vivo* OP exposure models to better understand the potential cause-effect relationship.

Multiple behavioral tests can be used to assess learning and memory in animal models, including the Morris water maze (MWM), Barnes maze (BM), radial arm maze (RAM), novel object recognition (NOR), and operant conditioning (OC) tasks (Vorhees and Williams, 2014; Quillfeldt, 2016). MWM, BM, and RAM tasks rely on hippocampal function to remember an escape or reward location based on association with spatial cues. MWM is more aversive than BM, as the animal must swim to escape instead of walking on an open platform in search of the escape hole in the BM. RAM uses a multi-armed platform to test the animal's ability to locate a reward on a single arm, which is indicated by a spatial cue. The NOR task examines recognition memory by exploiting the propensity of rodents to spend more time exploring a novel object than a familiar one. Finally, OC tasks, either passive or active avoidance, use operant training procedures in which an animal's response is learned and elicited based on a predictable aversive stimulus. Each paradigm has its own set of advantages and disadvantages, and these characteristics should be carefully considered when selecting which task to apply in the laboratory. For a review on the advantages and disadvantages of specific behavior tasks please see: (Vorhees and Williams, 2014; Quillfeldt, 2016). Described below are studies that have examined the impact of OP exposure relevant to occupational exposures in humans on learning and memory tasks in animal models.

Executive functions of learning and memory are impaired in humans and rodents following prolonged or chronic, low to moderate CPF exposure (Farahat et al., 2003; Rohlman et al., 2007; Ismail et al., 2012). For example, rats exposed to CPF for 14 days at levels producing 30–60% plasma ChE inhibition 24 h after single injection (2.5, 10, 18, and 25 mg/kg/d) exhibited deficits in the MWM (Terry et al., 2003). During the learning phase of the task, rats exposed to CPF at 18 or 25 mg/kg required significantly longer time to learn the location of the escape platform. Rats exposed at these levels also exhibited memory deficits, spending a lower percentage of their time in the target escape quadrant and crossing the location of the target fewer times than controls during the probe test of memory. Although swim speeds were decreased in the high CPF treatment groups, this cannot solely account for learning and memory deficits as all groups were able to perform the visible platform task equally. Furthermore, a second group of rats exposed to 25 mg/kg/d CPF for 14 days, which produced approximately 60% plasma ChE inhibition, were tested in the MWM following a 14-day washout period to determine whether the deficits persisted longer than 1 week. Results showed that CPF-exposed rats performed normally in the MWM, indicating that CPF-induced deficits in this exposure paradigm were transient (Terry et al., 2003). Interestingly, longer CPF exposure paradigms cause greater suppression of plasma ChE over time, but similar deficits in MWM. Rats exposed to CPF at 18 mg/kg every other day for 30 days exhibited 80% plasma ChE inhibition and deficits in MWM following a 7-day washout period (Terry et al., 2007). Even more severe and longer lasting deficits in learning and memory have been observed in both a modified RAM and MWM following prolonged exposure of rats to CPF. For example, exposure at 10 and 18 mg/kg CPF every other day for 30 days, causing plasma ChE suppression by up to 80%, resulted in RAM and MWM deficits even after prolonged washout periods of 50 and 140 days, respectively (Terry et al., 2012). Taken together, these studies support CPF-induced alterations in learning and memory, with length of exposure influencing the persistence of the deficit. Of note, these studies were performed in the same laboratory, and results would be strengthened with confirmation by an independent laboratory.

Although DDVP has not been as extensively studied as CPF, one study supports an effect of this agent on learning and memory. Specifically, animals exposed to DDVP daily for 12 weeks at 1 and 6 mg/kg/d, producing 10–55% reductions in serum ChE and 20–80% reductions in brain AChE, performed significantly worse in a dose-dependent manner on MWM and OC passive avoidance tasks (Verma et al., 2009). It is possible that MWM learning deficits could be attributed to deficits in locomotor activity, as indicated by deficient performance in the rotatod task. However, deficient motor activity is less likely to influence the outcome of the passive avoidance OC task, in which animals still displayed cognitive deficit. Interestingly, although exposure to DDVP for 12 weeks at 1 mg/kg/d produced learning and memory deficits, it did not produce significant decreases in ChE activity, while exposure to the higher DDVP dose did. These findings suggest that DDVP may cause behavioral deficits independent of AChE inhibition.

DFP, a highly toxic OP with a relatively low LD_50_ (refer to Table 5), induces learning and memory deficits in rodent exposure models at doses that do not trigger cholinergic crisis. For example, Bushnell et al. observed cognitive deficits in rats repeatedly exposed to 0.1 or 0.2 mg/kg DFP for 21 days, yielding approximately 75% reductions in AChE activity in the hippocampus and prefrontal cortex but unaccompanied by cholinergic crisis or overt toxicity (Bushnell et al., 1991). DFP-exposed animals performed poorly relative to controls in OC testing with choice and matching accuracy, indicating impaired learning and memory. However, the authors also observed significant impairment of mobility in animals exposed to DFP, which complicates interpretation of performance in this behavioral learning task. After a 3–4 week washout period, behavioral deficits were not observed and mobility also returned to normal, demonstrating reversibility of the toxicity. In addition, rats exposed to 0.05 and 0.25 mg/kg/d DFP for 14 days, producing up to approximately 50% AChE enzyme activity inhibition in the hippocampus and frontal cortex, exhibited learning and memory deficits in the MWM in a dose-dependent manner (Prendergast et al., 1997). Effects on learning and memory are further supported by a subsequent study in which rats exposed to DFP at 0.25 mg/kg/d for 14 days exhibited spatial learning and memory deficits in the MWM (Prendergast et al., 1998). Interestingly, long-term recall learning (or relearning) in subsequent MWM testing was not impaired, and neither was performance in delayed discrimination testing, a form of OC learning. In another study by Terry et al. (2011), DFP-exposed rats demonstrated impairment in the MWM and NOR tasks following 0.25, 0.5, and 0.75 mg/kg DFP exposure every other day for 30 days, producing roughly 55–80% reductions in plasma ChE activity. However, DFP exposure did not elicit sensorimotor deficits following a 13-day washout period, as measured by auditory startle response (ASR) task and prepulse inhibition (PPI) (Yeomans, 1995). These measurements are used to gauge a phenomenon in which a weaker prepulse (stimulus) inhibits the reaction to a subsequently stronger pulse (stimulus), and serve as commonly used correlates of pre-attentive stages of information processing. Persistent effects of this type of exposure were observed in another study in which exposure to 0.75 mg/kg DFP every other day for 30 days, producing up to 75% reductions in plasma ChE activity and 85% reductions in brain ChE activity, produced deficits in the MWM that persisted following a 140-day washout period (Terry, 2012). However, a similar effect of DFP exposure on the RAM was not observed, for reasons that remain unclear.

**Table 5:** **LD_**50**_ (dose lethal to 50% of the test sample) information for selected OPs**.

**OPs and LD**_**50**_**s**					
**OP**	**Mouse Oral LD_50_ (mg/kg)**	**Mouse Dermal LD_50_ (mg/kg)**	**Rat Oral LD_50_ (mg/kg)**	**Rat Dermal LD_50_ mg/kg**	**Toxicity in Humans**
Acephate	361^A^		1030–1447^A^		Moderate^B^
Azinphos-methyl	8–20^C^	65^C^	4^C^	88–220^C^	High^B^
Chlorpyrifos	60^D^	200^D^	96–270^D^	>2000^E^	Moderate^B^
Diazinon	80–135^F^		300–1250^G^		Moderate^B^
Dichlorvos	61–175^C^^,^^H^	206^C^	50 < 500^C^^,^^H^	70.4–250^C^^,^^H^	Moderate^B^
Dimethoate	160^C^		30–330^I^	100–2000^C^^,^^I^	Moderate ^B^
Disulfoton			2–12^J^	3.6–15.9^J^	High^B^
Ethoprop			61.5^K^		Moderate^B^
Fenamiphos	22.7^H^		2–24.8^H^	72–154^H^	High^B^
Malathion	400–4000^C^		1000–5500^C^	>4000^C^	Low^B^
Methamidophos			16–21^G^	50^G^	High^B^
Methidathion	18–25^C^^,^^G^		25–54^C^^,^^G^	85–94^C^^,^^G^	Moderate^B^
Methyl Parathion	14.5–19.5^C^^,^^G^	1200^C^^,^^G^	6–50^C^^,^^G^	67^C^^,^^G^	High^B^
Naled	330–375^D^		91–430^D^	800^D^	Moderate^B^
Oxydemeton-methyl			50^L^	85^L^	Moderate^B^
Phorate	2.25–6.59^C^		1.1–4^C^	2.5–6.2^C^^,^^G^	High^B^
Phosmet	23.1–50.1^M^		113–316^M^		Moderate^B^
Profenofos	298^N^		358–1178^N^	300–2000^N^	Moderate^B^

A *Worthing et al., 1987*.

B *Roberts and Reigart, 2013*.

C *Gallo and Lawryk, 1991*.

D *Meister, 1992*.

E *Racke, 1992*.

F *World Health Organization, 2009*.

G *Kidd and James, 1991*.

H *U.S. Public Health Service, 2016*.

I *Cheminova Agro A/S, 1991*.

J *U.S. Environmental Protection Agency, 1984*.

K *Powers, 1965*.

L *Sax, 1984*.

M *Food and Agriculture Organization of the United Nations, 1978*.

N *Food and Agriculture Organization of the United Nations, 2011*.

While MP exposure has been strongly linked to carcinogenicity and peripheral axonopathy, studies also indicate MP's potential to induce cognitive deficits. For example, exposure to MP at 2 mg/kg/d for 10 days reduced plasma ChE activity by 30% and induced learning deficits in the MWM in rats (Castillo et al., 2002). Further examination of MP-induced learning deficits by Sun et al. (2006), however, found that exposure of rats to 3 mg/kg/d MP for 21 days reduced brain region-specific ChE activity by up to 80%, yet produced no deficits in OC active avoidance conditioning. In this task, an animal is conditioned to respond to aversive stimuli, generally in the form of sound accompanied by delayed foot shock. Movement of the animal to a safe location to avoid the shock following the sound indicates learning (Zovkic and Sweatt, 2013). Exposed animals were not impaired in either learning or memory. Discrepancies between these two studies pertaining to the cognitive effects of MP could be related to differences in behavioral testing, but are unlikely to involve the duration or dose of exposure, both of which were lower in the study conducted by Castillo et al. (2002).

Finally, repeated, low- to moderate-level malathion exposure can also produce learning and memory deficits with minimal brain AChE inhibition. Specifically, exposure to malathion for 15 days at 30 and 100 mg/kg/d, causing 40% hippocampal AChE inhibition after 15 days with 100 mg/kg/d dosing, produced deficits in the object location task, a modified NOR task that depends on the spatial memory of the animal (dos Santos et al., 2016). Exposed rats spent significantly less time with the displaced object than controls, indicating acquisition of memory deficits, which was observed in the absence of changes in exploratory behavior or locomotive activity.

In conclusion, a substantial body of literature indicates that OP exposure is associated with deficits in cognition, as studies have shown impairments in both learning and memory aspects of behavioral tasks. Thus, OP exposure appears to disrupt both acquisition of skills as well as expression of retention of these learned skills.

### Attention and impulsivity

In addition to learning and memory impairments, epidemiological evidence suggests that OP exposure may induce changes in attention and impulsive behavior in adults (Farahat et al., 2003; Rohlman et al., 2007; Ismail et al., 2012; Meyer-Baron et al., 2015; Muñoz-Quezada et al., 2016). Attention and impulse control are critical aspects of executive function that play fundamental roles in information processing and are also impaired in numerous psychiatric disorders. Deficits in attention and impulsive behavior have been observed in animal models of repeated, low to moderate OP exposures. For example, Middlemore-Risher et al. (2010) observed disturbances in sustained attention of rats exposed every day for 14 days, or every other day for 30 days, to 18 mg/kg of CPF, reducing plasma ChE by approximately 70–80% and brain region-specific AChE by 25–60% at the final day of exposure (Middlemore-Risher et al., 2010). The authors reported increased impulsivity, as measured by the five Choice-Serial Reaction Time Task (5C-SRTT) that requires the test subject to correctly identify a temporarily illuminated opening in order to receive a reward. The length of time in which the opening is illuminated influences the required attention and impulse control of the test subject (Hayward et al., 2016). In support of attention impairments, deficits in PPI were also observed in CPF-exposed groups (Middlemore-Risher et al., 2010). Interestingly, PPI deficits appeared to be transient following a 30-day washout under both the 14- and 30-day exposure paradigms. Following the washout period, plasma ChE returned to 80 and 89% of normal, respectively, and brain AChE activity returned to normal in every region tested for both exposure paradigms, except in the striatum and basal forebrain.

Studies have also shown that DFP can influence attention and impulsive behavior. Rats exposed to 0.50 mg/kg DFP every other day for 30 days, inducing 40% plasma ChE activity inhibition at the end of the exposure period, exhibited impaired accuracy in the 5C-SRTT (Terry et al., 2014). Furthermore, these exposures increased the number of premature, and therefore impulsive, responses to the challenge as well as timeout responses taking place after incorrect or premature responses. These effects were greatest during the exposure period, but still modestly present after a 45-day washout period (Terry et al., 2014). Interestingly, previous studies using this same exposure paradigm (every other day for 30 days) at 0.25, 0.50, 0.75, and 1.0 mg/kg DFP did not produce deficits in PPI following a 13-day washout (Terry et al., 2011). These results are consistent with the findings by Middlemore-Risher et al. (2010) regarding the transient effects of CPF on attention.

### Motility and motor coordination

A common manifestation of acute OP exposure in humans is organophosphate ester-induced delayed neuropathy (OPIDN). OPIDN is associated with the ability of OPs to inhibit neuropathy target esterase (NTE), an alternative OP-target to AChE. OPIDN can occur with or without cholinergic crisis and other acute toxicities, as well as in the case of some chronic exposures, and is characterized by degeneration of distal portions of long axons within 1–4 days of exposure. Clinical manifestations of OPIDN include weakness, alterations in reflexes, limb tingling, loss of sensation, and impairments in locomotion and coordination (Richardson, 1995; Jamal, 1997; Lotti and Moretto, 2005). Several OPs have been linked to movement and coordination deficits in humans following occupational exposures, including CPF, DFP, DDVP, parathion, and T_*O*_CP (Lotti and Moretto, 2005; Ehrich and Jortner, 2010). Here, we discuss the laboratory evidence concerning OPs and induced neuropathies or motor and coordination deficits at low to moderate, repeated exposures.

Studies conducted by Sun et al. (2006) revealed motor deficits in rat exposed to sub-lethal levels of MP, an OP known to induce neuropathies in humans. Rats administered 3 mg/kg/d MP for 21 days exhibited brain region-specific ChE activity reductions by 80% and showed decreases in basal locomotor activity. These deficits included decreases in total distance traveled, vertical activity, ambulation time, and stereotyped time. Furthermore, exposed animals displayed poorer performance on rotarod testing, which assesses fine motor coordination. It should be noted, however, that although exposure to MP at these levels did not produce overt cholinergic toxicities, animals did manifest early signs of cholinergic crisis, including purposeless chewing and irritability.

Rats exposed daily to 0.25 mg/kg DFP for 14 days, which produced 50% AChE activity inhibition in the hippocampus and frontal cortex, exhibited disturbances in spontaneous locomotion during open field testing (Prendergast et al., 1997). Interestingly, rats exhibited hyperactivity following exposure to lower levels (0.05 mg/kg/d) of DFP (AChE activity not measured), but conversely exhibited significant decreases in activity following exposure to higher DFP doses (0.25 mg/kg/d). Animals did not exhibit overt cholinergic crisis during either exposure. Furthermore, Terry et al. (2011) described deficits in the grip strength of rats exposed repeatedly to 1.0 mg/kg/d DFP, a dose that produced up to 80% plasma ChE inhibition. These effects appeared to be transient, as animals regained grip strength following a 14-day washout period. No exposure-related deficits in rotarod performance were observed. Finally, Bushnell et al. (1991) described reversible motor deficits in rats exposed to 0.1 and 0.2 mg/kg/d DFP for 21 days, causing 50–75% reductions in AChE activity in the hippocampus and prefrontal cortex. These deficits were assessed by locomotor activity in a delayed matching-to-position task, a learning and memory task that requires substantial locomotor activity (Rothblat and Kromer, 1991).

Chronic exposure to DDVP at 1 and 6 mg/kg/d for 12 weeks, producing 10–55% reductions in serum ChE and 20–80% reductions in brain AChE activities, significantly impaired motor coordination and muscle strength in rats (Verma et al., 2009). DDVP-exposed groups performed significantly worse than controls on rotarod testing. These findings were reproduced by exposing rats to 2.5 mg/kg/d DDVP for 12 weeks, producing no significant reductions in substantia nigra or corpus striatum brain AChE activity (Binukumar et al., 2010). DDVP-exposed rats exhibited decreased locomotor activity in open field analysis at 6 and 12 weeks, as measured by decreased distance traveled and increased immobile time. Again, DDVP-exposed rats performed poorer during rotarod testing when compared to controls at 6 and 12 weeks (Binukumar et al., 2010).

In addition, Terry et al. (2003) described deficits in rat hindlimb, and to a lesser extent forelimb, grip strength after exposure to 2.5 mg/kg/d CPF for 38 days, which produced 30% plasma ChE inhibition 24 h following a single injection. Here, ChE levels were not measured at the end of the exposure period. Effects on grip strength were abated during a 5-day washout period, and animals appeared normal on day 6. Conversely, no deficits in motor activity were observed when adolescent rats (postnatal day 27–36) were exposed to 2.5, 5, 10, or 20 mg/kg/d CPF for 10 days, indicating that longer exposure periods may be required to induce deficits in motility (Chen et al., 2014).

### Depression and anxiety

Some epidemiological studies suggest that occupational exposures to OPs induce changes in the emotional state of individuals, based on correlations between occupational exposures and depression, anxiety, and suicide (Parrón et al., 1996; Lee et al., 2007; Beseler et al., 2008; Freire and Koifman, 2013; Beard et al., 2014). A meta-analysis conducted in 2013 on occupational exposure and psychiatric issues, however, suggests these epidemiological studies are limited and inconclusive (Freire and Koifman, 2013). *In vivo* studies examining these endpoints in preclinical models are challenging to interpret, as emotional affect is not known to be exhibited by rodents. Instead, preclinical models constitute rodent behavioral outcomes related to human behaviors driven by the affective state. Thus, behavioral tasks such as elevated-plus maze (EPM), novelty-suppressed feeding test, Porsolt forced swim task (FST), learned helplessness, and exploratory behavior offer behavioral correlates of human depression and anxiety (Bailey and Crawley, 2009). In the EPM, animals are allowed to explore an elevated maze containing two closed (protected) arms and two open (unprotected) arms. Animals spending higher percentages of time on the open arms are either considered hyperactive or to be participating in risky behaviors, while animals not exploring the open arm are considered to be exhibiting anxiety-like or risk-aversive behaviors. Novelty-suppressed feeding relies on the fact that a novel environment can suppress feeding behaviors in rodents. Following a restricted feeding paradigm, the animal is placed into a novel environment and given the choice to explore the center of an arena where food is located. Animals that are reluctant to ambulate to the food are considered to exhibit anxiety-like behaviors. The FST and learned helplessness tasks more specifically measure depression-like phenotypes, and both tasks rely on the efforts of the animal to escape a stressful situation (Yan et al., 2010). Finally, exploratory behavior can be examined in almost all of the above-described tasks, and is dependent on affect (Yan et al., 2010).

Adolescent rats (postnatal day 29) exposed to CPF for 7 consecutive days to 10, 20, and 40 mg/kg/d showed alterations in affect-related behavior (Chen et al., 2011). Animals treated with 10 and 20 mg/kg/d CPF did not exhibit any differences in the EPM when compared to control animals, but 40 mg/kg/d CPF was sufficient to induce the risky behavior of more time spent on the open arm of the EPM. In addition, animals exposed to 10 mg/kg/d CPF showed an increase in immobility time in the FST, while 20 and 40 mg/kg/d doses did not induce changes in behavior. Finally, all exposures to CPF in this paradigm resulted in deficits in the novelty-suppressed feeding task, indicating anxiety-like behavior (Chen et al., 2011). Unfortunately, ChE was not measured in this study, and because the exposure paradigm was shorter and the rats were adolescent, it is difficult to relate this study to others. However, an additional study examined the depression-like phenotype of adolescent rats (postnatal day 27–36) exposed to 2.5, 5, 10, or 20 mg/kg/d CPF for 10 days (Chen et al., 2014). Here, the 10 mg/kg/d CPF exposure increased immobility in FST, while 5, 10, and 20 mg/kg/d CPF exposures all induced increased escape failures in the learned helplessness task, indicating greater susceptibility to depression-like phenotypes. Importantly, these CPF-induced changes in behavior were not accompanied by deficits in locomotor activity. Again, ChE was not reported, making it difficult to relate this study to previously discussed studies. However, the CPF doses were comparable to those used by Terry et al. (2003), which produced 30–60% plasma ChE inhibition 24 h following 2.5, 10, 18, and 25 mg/kg single injections (Terry et al., 2003).

Finally, exploratory behavior, such as distance traveled, sniffing, rearing, and forward elongation of the head can be used to assess the animal's general well-being, and is thus thought to correlate to human affective experience (Harro, 1993). It has been repeatedly shown that in nonthreatening scenarios, animals spend more time moving throughout the environment interacting with unfamiliar objects. By contrast, an animal placed in a threatening scenario will typically exhibit freezing behavior or cower in a single position. Accordingly, exploratory behavior has been used to describe innate behavioral movements, fear responses, brain injury, and response to various pharmacological agents (Harro, 1993).

Rats exposed to DFP at 0.25 mg/kg/d for 14 days exhibited decreased rearing and sniffing exploratory activity (Prendergast et al., 1998). DFP-exposed animals exhibited tolerance after a week of testing, at which point their exploratory behavior was comparable to controls. Furthermore, deficits in vertical and horizontal activity were observed in open field analysis of animals exposed to 0.75 mg/kg/d DFP every other day for 30 days, producing up to 75% reductions in plasma ChE activity and 85% reductions in brain ChE activity (Terry et al., 2012). Other OPs also alter exploratory behavior. For example, CPF exposure at 2.5, 10, 18, and 25 mg/kg/d for 14 days reduced plasma ChE from 30 to 60% following a single injection and also produced decreases in sniffing and rearing behaviors (Terry et al., 2003). Additionally, 12 weeks of exposure to 2.5 mg/kg/d DDVP resulted in no significant ChE inhibition in the brain and produced decreases in rearing behaviors during open field analysis when compared to controls (Binukumar et al., 2010). Conversely, 15-day malathion exposure at 30 and 100 mg/kg/d caused 40% hippocampal AChE inhibition and did not produce changes in exploratory activity, as measured by rearings in the open field (dos Santos et al., 2016).

## Mechanisms of chronic OP neurotoxicity

The molecular mechanisms by which chronic exposure to OPs causes neurobehavioral deficits remain speculative. It is well-established that OPs phosphorylate the active site of AChE to inhibit its enzymatic activity, resulting in accumulation of synaptic acetylcholine and altered cholinergic signaling. However, not all OPs precipitate the same clinical manifestations, and several OPs also induce symptoms that do not correlate with AChE inhibition (Pope, 1999; Costa, 2006; Rohlman et al., 2011). Therefore, it is widely postulated that targets in addition to AChE are involved in OP-induced neurotoxicity. For a full review on potential noncholinergic molecular targets, (see Pope, 1999; Costa, 2006; Jett and Lein, 2006; Terry, 2012). Furthermore, tolerance to AChE enzyme inhibition is often achieved with prolonged exposure due to down-regulation of muscarinic and nicotinic acetylcholine receptors linked to overstimulation of cholinergic pathways (Schwab et al., 1981; Albuquerque et al., 1985; Bushnell et al., 1991, 1993). In order to more completely understand the molecular underpinnings of OP-induced neurobehavioral deficits, various reductionist models have been exploited to study the molecular effects of OPs in relevant cell types. Some studies have also investigated molecular changes following *in vivo* exposures, and attempted to correlate these findings with behavioral outcomes. Here, we summarize studies that examine the effects of OP exposures that do not induce overt toxicity or significantly inhibit ChE activity in isolated tissue or cell culture systems. We also summarize published attempts to reproduce prolonged, low- to moderate-dose occupational exposure parameters *in vitro*, as well as *in vivo* studies that investigate potential molecular mechanisms of neurotoxicity. These findings are presented with respect to the following six categories of molecular mechanisms of OP neurotoxicity: cytotoxicity, aberrant neuronal cytoarchitecture, aberrant energy homeostasis, aberrant neurotransmission, neuroinflammation, and impairment of the blood brain barrier. See Table 6 for a summary of the following findings.

**Table 6:** **Summary of molecular and cellular endpoints altered by OPs in experimental models of occupational organophosphate exposure**.

**Endpoint**		**OP**	**Direction of change**	**Model system**	**Publication**
Cytotoxicity (1)	Cell death (unspecificed)	CPF	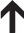	*In vivo* (Wistar rats)	Prendergast et al., 2007
		DZ	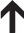	*In vitro* (primary culture)	Rush et al., 2010
		Parathion	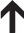	*In vitro* (primary culture)	Berríos et al., 2015
		DDVP	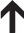	*In vivo* (Wistar rats)	Binukumar et al., 2010
			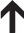	*In vivo* (Wistar rats)	Binukumar et al., 2011
	Cell death (apoptotic)	DDVP	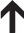	*In vivo* (Wistar rats)	Kaur et al., 2007
		Malathion	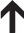	*In vivo* (Swiss albino mice)	dos Santos et al., 2016
	Cell death (excitotoxic)	CPF	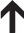	*In vitro* (primary culture)	Rush et al., 2010
	Cell death (necrotic)	Parathion	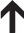	*In vitro* (primary culture)	Yousefpour et al., 2006
	Neuron density	CPF	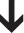	*In vivo* (Swiss albino mice)	Mitra et al., 2009^*^^#^
			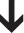	*In vivo* (Swiss albino mice)	Lim et al., 2011^*^
Cytoarchitecture (2)	Axonal Outgrowth	CPF	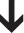	*In vitro* (primary culture)	Howard et al., 2005
			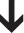	*In vitro* (primary culture)	Yang et al., 2008
	Dendritic Outgrowth	CPF	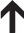	*In vitro* (primary culture)	Howard et al., 2005
			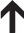	*In vitro* (primary culture)	Yang et al., 2008
	Neurite Outgrowth	DZ	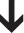	*In vitro* (primary culture)	Pizzurro et al., 2014a
			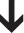	*In vitro* (primary culture)	Pizzurro et al., 2014b
		Parathion	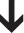	*In vitro* (primary culture)	Yousefpour et al., 2006
			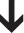	*In vitro* (primary culture)	Berríos et al., 2015
		T*o*CP	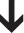	*In vitro* (primary culture)	Chen et al., 2013
			−	*In vitro* (primary culture)	Duarte et al., 2016
			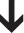	*In vitro* (primary culture)	Hausherr et al., 2014
	Synaptic Density	CPF	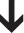	*Ex vivo* (C57B1/6J slice culture)	Speed et al., 2012
Energy homeostasis (3)	ETC	DDVP	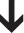	*In vivo* (Wistar rats)	Kaur et al., 2007
	Mitochondrial Integrity	CPF	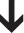	*In vitro* (primary culture)	Middlemore-Risher et al., 2011
	Mitochondrial Activity	T*o*CP	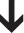	*In vitro* (primary culture)	Duarte et al., 2016
	Oxidation Pathways	DZ	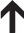	*In vitro* (primary culture)	Pizzurro et al., 2014b
			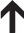	Isolated synaptosomes (rat)	Colovic et al., 2015
		DDVP	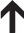	*In vivo* (Wistar rats)	Kaur et al., 2007
			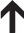	*In vivo* (Wistar rats)	Binukumar et al., 2010
			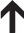	*In vivo* (Wistar rats)	Binukumar et al., 2011
		Malathion	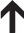	*In vivo* (Swiss albino mice)	dos Santos et al., 2016
Neurotransmission (4)	Axonal transport	CPF	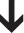	*In vivo* (Wistar rats)	Terry et al., 2003
			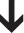	*In vivo* (Wistar rats)	Hernandez et al., 2015
		DFP	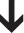	*In vitro* (primary culture)	Gao et al., 2016
	Motor proteins	CPF	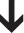	Isolated kinesin and microtubules	Gearhart et al., 2007
			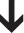	*In vivo* (Wistar rats)	Prendergast et al., 2007
			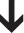	Isolated kinesin and microtubules	Grigoryan and Lockridge, 2009
			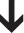	*In vitro* (primary culture)	Middlemore-Risher et al., 2011
		DFP	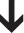	Isolated kinesin and microtubules	Gearhart et al., 2007
			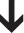	*In vitro* (primary culture)	Gao et al., 2016
	Neuropeptide regulation	CPF	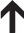 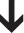	*In vivo* (Long Evans rats)	Lee et al., 2016
			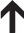 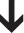	*In vitro* (PC12 cells)	Slotkin and Seidler, 2010
		DZ	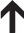 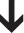	*In vitro* (PC12 cells)	Slotkin and Seidler, 2010
	Ca^2+^ signaling	CPF	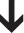	*In vitro* (PC12 cells)	Meijer et al., 2014
			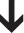	*In vitro* (PC12 cells)	Meijer et al., 2015
		Parathion	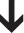	*In vitro* (PC12 cells)	Meijer et al., 2014
		T*o*CP	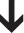	*In vitro* (primary culture)	Hausherr et al., 2016
	Receptor downregulation	CPF	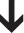	*In vivo* (Sprague-Dawley rats)	Huff et al., 2001
		DFP	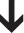	*In vivo* (Long Evans rats)	Mundy et al., 1993
		MP	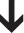	*In vivo* (Sprague-Dawley rats)	Ma et al., 2003
			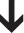	*In vivo* (Sprague-Dawley rats)	Sun et al., 2003
	Receptor blocking	Parathion	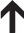	*In vitro* (primary culture)	Rocha et al., 1996
	Electrophysiology	Parathion	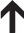	*In vitro* (primary culture)	Rocha et al., 1996
		CPF	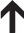	*In vivo* (Wistar rats)	Muller et al., 2014
			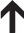 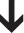	*Ex vivo* (C57B1/6J slice culture)	Speed et al., 2012
	Signal transduction	CPF	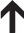 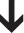	*In vivo* (Wistar rats)	Muller et al., 2014
		DFP	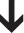	*Ex vivo* (Wistar rats)	Terry et al., 2011
		T*o*CP	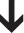	in vitro (primary culture)	Hausherr et al., 2016
Neuroinflammation (5)	Cytokine regulation	CPF	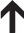	*In vitro* (Human fetal astrocytes)	Mense et al., 2006
	Microglia activation	CPF	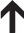	*In vivo* (Swiss albino mice)	Lim et al., 2011^*^
		DDVP	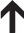	*In vivo* (Wistar rats)	Binukumar et al., 2011
		Malathion	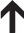	*In vivo* (Swiss albino mice)	dos Santos et al., 2016
Blood brain barrier (6)	BBB integrity	CPF	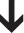	*In vitro* (BMEC and primary culture)	Parran et al., 2005
		Malathion	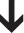	*In vitro* (RBE4 cells)	Balbuena et al., 2010
	BBB maintanence proteins	Malathion	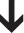	*In vitro* (RBE4 and BMEC cells)	Balbuena et al., 2011

* Indicates route of exposure other than SC (topical or tail patch);

# *Indicates test subjects were female*.

*PC12, adrenal pheochromocytoma cell line; BBB, blood brain barrier; BMEC, bovine brain microvascular endothelial cell line; CPF, chlorpyrifos; DDVP, dichlorvos; DFP, diisopropyl-fluorophosphate; DZ, diazinon; ETC, electron transport chain; MP, methyl parathion; RBE4, rat brain microvascular endothelial cell line; ToCP, tri-ortho-cresyl-phosphate*.

### Cytotoxicity

Many studies have demonstrated that OPs induce neuron damage or death at levels that do not ablate ChE activity. Cytotoxic effects are concentration-dependent, and in certain cases, such as CPF or parathion, differentially mediated by the parent compound vs. the metabolite. For example, 0.1, 1, and 10 μM chlorpyrifos-oxon (CPO), a CPF metabolite that is a potent ChE inhibitor, produces neuronal injury in the CA1 and CA3 regions of the hippocampus (Prendergast et al., 2007). Hippocampal slice cultures treated with CPO at levels that reduced hippocampal AChE activity by 50% after 3 and 7 days of exposure also exhibited neuronal injury that was both concentration- and time-dependent, as measured by propidium iodide fluorescence (PI), a polar fluorescent dye that penetrates damaged cell membranes to bind to nucleic acids within the cell (Zimmer et al., 2000). CPF exposure at 100 μM and CPO exposure at 1, 10, 30, and 100 μM induced neuronal injury via excitotoxic mechanisms (Rush et al., 2010). In this cortical culture system, 100 μM CPF inhibited AChE by 80%, and 1, 10, 30, and 100 μM CPO inhibited AChE by 80–90%, after 24 h. Pharmacologic antagonism of glutamate receptors significantly attenuated the neurotoxic effects of CPF and CPO, suggesting that CPF induces excitotoxic neuronal death. Subchronic CPF exposure also decreases neuron density *in viv*o. Mice exposed topically for 18 days to 40.4 and 101 mg/kg CPF, which produced 75–95% serum ChE inhibition and 55% brain AChE inhibition (40.4 mg/kg/d produced no significant brain AChE inhibition), showed dose-dependent decreases in hippocampal neuron cell counts (Mitra et al., 2009). When CPF-exposed mice were concomitantly stressed by 6 min of forced swimming in 38°C water, not only was AChE inhibition exacerbated, but greater reductions in hippocampal neuron cell counts were noted as well. In addition, when mice were topically exposed to 20.2 and 40.4 mg/kg/d CPF for 7 days, which inhibited serum ChE by 30%, significant decreases in hippocampal neuron cell density and increased astrocyte reactivity (only at 40 mg/kg/d) were apparent (Lim et al., 2011). Interestingly, concurrent stress exposure of 6 min of forced swimming in 38°C water did not exacerbate the effects of CPF exposure in this study. The authors suggest that shorter exposure time and lower doses of exposure could be responsible for the discrepancy in stress exacerbation between the two studies (Lim et al., 2011).

DZ (Rush et al., 2010), parathion (Yousefpour et al., 2006; Berríos et al., 2015), and DDVP (Binukumar et al., 2010; Binukumar and Gill, 2010) have also been shown to induce cytotoxicity following exposures that do not inhibit ChE activity *in vitro*, or exposures that do not cause overt toxicity *in vivo*. DZ produced neuronal cell death in primary cortical neurons following 24 h of exposure to 30 and 100 μM, resulting in approximately 50% AChE inhibition (Rush et al., 2010). Interestingly, no cell death was observed following exposure of primary cortical neurons to levels of the DZ metabolite diazinon-oxon (DZO) that produced 60–90% AChE inhibition. These results suggest that AChE inhibition does not play an integral role in cell death. Furthermore, Yousefpour et al. (2006) observed dose- and time-dependent changes in cell viability, cell expansion, and cellular morphology of primary hippocampal cultures following a single exposure to the parathion metabolite, paraoxon (POX), at 10, 30, 40, 50, 100, and 150 μM. One week following POX exposure at 30, 40, 50, and 100 μM, cell viability was significantly reduced, as measured using Neutral Red (NR) staining, a dye that is normally incorporated into intact lysosomes of healthy cells (Repetto et al., 2008). Three weeks after POX exposure, 10 μM concentrations also decreased cell viability, and both the 30 and 100 μM groups showed cellular blebbing. The 100 μM group also showed cytoplasmic volume decrease and increased number of vacuoles, characteristics of necrotic cell death (Yousefpour et al., 2006). This data was supported by a later study showing that POX exposure for 6 days at 50, 100, 150, 200, and 300 μM resulted in reduced cell viability of cultured neural stem and progenitor cells (dNPCs), as measured by MTT assay and PI (Berríos et al., 2015). Despite 200 μM POX exposures causing 50% AChE inhibition, the authors concluded that cell viability was unrelated to AChE inhibition, based on studies using pyridostigmine (PY), a reversible AChE inhibitor that did not reduce cell viability despite producing the same degree of AChE inhibition (Berríos et al., 2015).

*In vivo* OP exposure studies have also demonstrated cytotoxicity and cell death following repeated exposures. Rats treated with 6 mg/kg/d DDVP for 12 weeks exhibited activation of apoptotic pathways, with increases in cytochrome c release from mitochondria and activation of caspase-3, common markers of apoptosis (Kaur et al., 2007). Electron microscopy was used to confirm these findings, revealing characteristic morphological features of apoptosis in a separate cohort of exposed rats. In addition, rats exposed to 2.5 mg/kg/d DDVP for 12 weeks, a dose that produced no significant reductions in substantia nigra or corpus striatum brain AChE activity, exhibited 60–70% reductions in dopaminergic neurons of the substantia nigra (Binukumar et al., 2010). Of note, neuronal cell loss was not observed in the cortex of exposed animals. Also, malathion exposure upregulated apoptotic pathways in the hippocampus *in vivo*, as measured by expression levels of the apoptotic signaling proteins Bax and Bak (dos Santos et al., 2016). Stereological cell counts were not obtained, but choline acetyltransferase protein expression was unaffected. Thus, the authors concluded that despite apoptotic pathway activation there was no neuronal cell loss in the hippocampus.

### Aberrant neuronal cytoarchitecture

Alterations in the cytoarchitecture or morphology of cells in the nervous system have been associated with diverse neurological symptoms. Changes in length, number, or branching patterns of axons or dendrites have been linked to psychiatric manifestations in patients, including depression, anxiety, AD, and PD (Marsden, 2013; Rubia et al., 2014; Canu et al., 2015; Negrón-Oyarzo et al., 2016). Axonal length, neurite length, neurite number, and dendritic branching in neuronal culture systems have also been shown to be altered following exposure to a variety of OPs, including CPF (Howard et al., 2005; Yang et al., 2008; Speed et al., 2012), DZ (Pizzurro et al., 2014a,b), parathion (Yousefpour et al., 2006; Berríos et al., 2015), and T_*O*_CP (Chen et al., 2013; Duarte et al., 2016; Hausherr et al., 2016). For example, in primary cultures of sympathetic neurons, CPF inhibited axon outgrowth but promoted dendritic arborization (Howard et al., 2005). The authors further demonstrated that the major metabolites of CFP, CPO, and trichloropyridinol (TCP), similarly have opposing effects on neuronal axons and dendrites. Specifically, CPO decreased overall axonal outgrowth, while CPF, CPO, and TCP each independently increased dendritic outgrowth. These effects were observed at concentrations that did not inhibit AChE and did not cause cytotoxicity. Furthermore, sympathetic neurons from superior cervical ganglia are not cholinergic, so in purified primary cultures the OP-mediated effect is due to non-cholinergic mechanisms. Further studies from this same lab examined the mechanism by which CPF and CPO inhibit axonal growth (Yang et al., 2008). The authors speculated that because the AChE protein has both enzymatic and morphogenic properties, CPF could interfere with the latter to elicit its effects on axonal growth. Sensory neurons cultured from the dorsal root ganglia of AChE^+/+^, AChE^+/−^, and AChE^−/−^ mice were employed to test this hypothesis. Using concentrations of CPF and CPO previously reported not to decrease cell viability or AChE activity, the authors demonstrated that these compounds only caused deficits in axonal outgrowth in cultures that expressed intact AChE. It was concluded that CPF and CPO decrease axonal growth by interfering with the morphogenic activity of AChE. In support of these findings, Speed et al. (2012) demonstrated that CPF decreased dendritic spine density *in vivo* following 5 days of exposure at 5 mg/kg/d, levels that inhibited hippocampal AChE by <10% after a single injection and by approximately 40% after 5 injections. Spine density was unaffected by CPF exposure when examined 1 week later. However, when spine density was re-examined 3 months after exposure, total spine numbers decreased relative to controls, which correlated with decreased hippocampal synaptic transmission (Speed et al., 2012). Although behaviors were not analyzed directly in these animals, similar dosing paradigms, as previously described, have produced learning and memory deficits correlating with these findings. Changes in hippocampal neuron numbers were not apparent at either the 1-week or 3-month time points. It is well known that morphological plasticity of both axons and dendrites are critical for the behaviors discussed above (Leuner and Gould, 2010; Marsden, 2013; Gipson and Olive, 2016). Therefore, it is possible that these changes could also occur in occupational OP exposures and contribute to neurobehavioral deficits.

DZ and its oxidized metabolite DZO also impair neurite outgrowth (Pizzurro et al., 2014a). Hippocampal neurons exposed for 24 h to 0.1, 1, and 10 μM DZ and DZO, which reduced AChE enzymatic activity by 50–80% at the highest concentrations, exhibited decreased neurite outgrowth in a dose-dependent manner, while the overall number of neurites per cell did not change. Concomitantly, indicators of oxidative stress were also increased with DZ and DZO. Hippocampal neurons were protected against the effects of DZ and DZO on neurite outgrowth in the presences of astrocytes, and the authors demonstrated that this was due to increased glutathione (GSH) levels (Pizzurro et al., 2014a). An additional study examined the relationship between hippocampal neurons and astrocytes following DZ and DZO exposure and found decreased neurite outgrowth, increased oxidative stress, and protective effects of astrocytes (Pizzurro et al., 2014b). Briefly, astrocytes were treated for 24 h with 0.1, 1, and 10 μM DZ and DZO, concentrations that did not affect cell viability and that reduced AChE activity by 20% at the highest concentrations of each OP. Hippocampal neurons cultured for 48 h were inverted on top of the previously OP-exposed astrocytes, and neurite outgrowth was measured. DZ and DZO at 10 μM inhibited neurite outgrowth and induced oxidative stress, as measured by dichlorofluorescein diacetate (DCF), a fluorescent dye that upon oxidization by reactive oxygen species (ROS) is converted to a dichlorofluorescein. Furthermore, astrocytic fibronectin was reduced following DZ and DZO 10 μM exposure, but fibronectin rescued neurite outgrowth deficits. The authors concluded that the effects on neurite outgrowth were caused by reductions in astrocytic fibronectin, a key component of the extracellular matrix crucial for cell adhesion, and that oxidative stress signals altered the interaction between hippocampal astrocytes and neurons (Pizzurro et al., 2014b).

Finally, studies have also shown the ability of parathion and T_*O*_CP to induce alterations in cytoarchitecture *in vitro*. Yousefpour et al. (2006) reported decreases in the number and length of neuronal processes in hippocampal primary cells 1 week after exposure to POX at 30, 40, 50, and 100 μM. These data were supported by an additional study showing that prolonged POX exposure at 16–66 μM POX indiscriminately decreased neurite outgrowth in dNPCs, as measured by Scholl analysis (Berríos et al., 2015). Additionally, T_*O*_CP dose-dependently inhibited neurite outgrowth in SH-SY5Y cells following 24 h treatment with 200, 500, and 1000 μM, and an autophagy inhibitor prevented and reversed this growth inhibition, indicating a critical role for autophagy in T_*O*_CP-induced neurite changes at high doses (Chen et al., 2013). At lower doses (10 μM), exposure to T_*O*_CP for 48 h did not decrease neurite number or outgrowth in rat primary cortical neurons, as measured by immunocytochemistry (Duarte et al., 2016). Esterase activity was also not significantly reduced under these exposure conditions. Conversely, Hausherr et al. (2014) reported that 10 μM T_*O*_CP exposure for 24 h reduced neurite area and branching in mouse primary cortical neurons (Hausherr et al., 2014). Differences in species or methods used to quantify neurites could be responsible for the discrepancies between these two studies. It is plausible that the number of neurites in the study by Duarte et al. (2016) were not affected, but that the morphology was altered. Alternatively, it is possible that the number of neurons in the study by Hausherr et al. (2014) was not altered, while the area of each neurite was reduced. Unfortunately, ChE levels were not examined in some of these studies, making it difficult to compare results between the different exposure paradigms.

### Aberrant energy homeostasis

Cellular metabolism is highly regulated to supply cells with ATP, and disturbances in energy homeostasis act as signals of cellular stress that trigger cell death (Tait and Green, 2010). Mitochondria are at the crux of energy homeostasis and cellular respiration. Damage to mitochondrial membrane potential can leave the cell vulnerable to injury and in some cases trigger apoptosis, while changes in the citric acid cycle can result in aberrant production of damaging ROS (Tait and Green, 2010). Mitochondrial dysfunction has been implicated in neurodegeneration (Migliore and Coppedè, 2009; McInnes, 2013), and several studies suggest that both deficiencies in mitochondrial function and oxidative damage caused by production of abnormal ROS can contribute to the pathogenesis of AD, PD, and ALS (Lin and Beal, 2006).

DDVP and DZ both elicit deleterious effects on cellular energy metabolism. For example, mitochondrial energy metabolism was impaired in rats following 12 weeks of exposure to DDVP at 6 mg/kg/d, via increasing Ca^2+^ uptake in mitochondria and altering important components of the electron transport chain (ETC), ultimately leading to apoptosis (Kaur et al., 2007). Although ChE activity was not reported in this study, work by Verma et al. (2009) using the same DDVP dose exposure paradigm reported 55% reductions in serum ChE activity and 80% reductions in brain AChE activity. The authors observed significantly increased Ca^2+^ uptake in mitochondria isolated from treated animals, as well as significant decreases in important enzymes in the ETC (Kaur et al., 2007). Furthermore, critical mitochondrial antioxidants, such as GSH and mitochondrial superoxide dismutase (Mn-SOD), were significantly depleted, while lipid peroxidation and protein oxidation were increased. The authors also found significant mitochondrial cytochrome c release and cleaved caspase-3, markers of apoptosis (Kaur et al., 2007). Exposure to DZ, and to a greater extent its oxidized metabolite DZO, has also been shown *in vitro* to affect energy homeostasis and oxidative stress pathways without reducing AChE activity. DZ and DZO induced oxidative stress in hippocampal neuron and astrocyte co-cultures in which astrocytes were treated for 24 h with 0.1, 1, and 10 μM DZ and DZO, concentrations that reduce AChE activity by 20% at the highest concentrations of each OP and did not affect cell viability (Pizzurro et al., 2014b). DZ and DZO increased the production of ROS in a concentration-dependent manner, as measured by DCF. Colovic et al. (2015) also examined expression levels of antioxidant enzymes, including catalase (CAT), SOD, and glutathione peroxidase (GPx) following DZ and DZO exposure. Here, isolated synaptosomes were exposed to DZ, DZO, and 2-isopropyl-6-methyl-4-pyrimidinol (IMP), a hydroxylated detoxified DZ metabolite, at increasing concentrations that did not alter synaptosomal integrity, as measured by lactate dehydrogenase (LDH) leakage (Colovic et al., 2015). AChE activity was measured in synaptosomes following addition of DZ, DZO, and IMP, and inhibition occurred in a dose-dependent manner with DZO causing the greatest AChE inhibition at 95%, and the highest concentration of DZO and IMP not affecting AChE activity. The authors found that DZ did not significantly alter activity of the antioxidant enzymes DZO and IMP at levels that affected AChE in entirely different capacities. However, these agents did induce activity of antioxidants, suggesting that alterations in antioxidant activity are not AChE-dependent. Specifically, DZO dose-dependently induced CAT, GPx, and SOD, while IMP dose-dependently induced SOD activity (Colovic et al., 2015).

*In vivo* exposure to DDVP and malathion also alters energy homeostasis. For example, DDVP induced oxidative stress after 12 weeks of exposure at 2.5 mg/kg/d, without significantly reducing substantia nigra (SN) or corpus striatum (CS) brain AChE activity (Binukumar et al., 2010). Significant increases in ROS levels and lipid peroxidation were also observed, while NADH dehydrogenase activity and cytochrome oxidase activity in the SN and CS decreased in exposed rats. Each of these outcomes indicates oxidative stress. In addition, malathion exposure decreased NADH dehydrogenase activity, but had no significant effects on Complex II or Complex IV of the ETC, following exposures that reduced hippocampal AChE by 0–40% over 15 days (dos Santos et al., 2016). Deficits in NADH dehydrogenase activity significantly correlated with observed deficits in the modified NOR behavioral task.

In addition to altering mitochondrial energy, OPs influence translocation of mitochondria within the cell. In neurons, mitochondria travel down the axon to provide energy throughout the axoplasm from the soma to the synapse. Middlemore-Risher et al. (2011) demonstrated that CPF and CPO influenced mitochondrial dynamics in primary cultured rat cortical neurons. Specifically, cells exposed to CPF and CPO contained mitochondria that were longer and moved less throughout the axon. Furthermore, there were significantly fewer mitochondria per axon in exposed groups. These changes occurred at concentrations that did not significantly inhibit AChE, as was the case for CPF, and also at levels that inhibited AChE by 50%, as was the case for CPO. The authors concluded that there were greater mitochondrial fusion events and fewer mitochondrial fission events following CPF exposure (Middlemore-Risher et al., 2011). In neurons, the balance of mitochondrial fusion and fission is critical for cell viability. Fission events aid in mitochondrial distribution within a cell, while fusion events allow mitochondria to repair and exchange material (Chen and Chan, 2009). It is interesting that deficits in ATP synthesis or increases in superoxide production were not observed in this study. Changes in mitochondrial dynamics could be caused by deficits in axonal transport, which have been reported following CPF exposure and are discussed below.

### Aberrant neurotransmission

Since the primary target of OPs is AChE, it is perhaps not surprising that other aspects of cholinergic signaling are also affected. For example, a well-established consequence of AChE inhibition by OPs is down-regulation of postsynaptic muscarinic acetylcholine receptors, which is thought to play a role in developing tolerance to chronic OP exposure (Schwab et al., 1981; Bushnell et al., 1991; Mundy et al., 1993; Stone et al., 2000; Huff et al., 2001; Ma et al., 2003; Sun et al., 2003; Costa et al., 2008). However, other OP-induced deficits in neurotransmission are also observed *in vitro*. For example, both DZ and CPF exposure change expression of genes coding for neuropeptides and their receptors in PC12 cells (Slotkin and Seidler, 2010). These changes occurred using concentrations that did not induce overt toxicity (as reported in Jameson et al., 2007), and were greater in magnitude in differentiating vs. undifferentiated PC12 cells. Immortalized cell lines, such as PC12 cells, are by necessity less biologically complex than primary cultured cells, and also have undergone genetic mutations that have rendered them immortal. These issues are important to consider when interpreting the biological effect, and rigorous verification of findings in immortalized cells should be conducted in both primary cultures and corresponding intact biological systems. Thus, it is encouraging that similar observations have been made using *in vivo* models of occupational OP exposure. Lee et al. (2016) found that repeated exposure to 3 and 10 mg/kg/d CPF produced 75–90% cortical AChE inhibition, 40–80% hippocampal AChE inhibition, and 90–100% whole blood ChE inhibition, respectively, at the end of a 21-day exposure period. These exposures induced transcriptomic changes in genes encoding for hippocampal neuropeptides in animals, including brain-derived neurotrophic factor (BDNF), cortistatin (CORT), and neuropeptide Y (NPY). Neuropeptides such as these are critical neurotransmission signals associated with diverse neurobehavioral deficits related to AD, PD, exploratory behavior, and anxiety- and depression-like phenotypes (Cortright et al., 1995; Heilig, 2004; Moran and Graeber, 2008; Borroni et al., 2009).

POX also disturbs gamma-Aminobutyric acid (GABA)-mediated neurotransmission, the major inhibitory neurotransmitter system in the brain. In cultured hippocampal cells, POX applied at 0.3–3 μM in the presence of atropine to block muscarinic receptor overstimulation modified the frequency of miniature postsynaptic currents (MPCs) without affecting peak amplitude or decay (Rocha et al., 1996). At higher concentrations of 30–300 μM, however, POX exposure reduced frequency, decay times, and peak amplitudes of GABA-mediated MPCs. Interestingly, POX exposure in the micromolar range additionally acted as an open-channel blocker, by noncompetitively and reversibly inhibiting GABA_A_, glycine, NMDA, and nicotinic acetylcholine receptors, events that would be expected to significantly alter neurotransmission within the brain.

Ca^2+^ signaling is critical for neurotransmission and neuronal depolarization events, which contribute to synaptic activity. Ca^2+^ ions activate ion channels and can act as second messengers in signal transduction pathways like G-protein coupled receptors. Thus, even small changes in Ca^2+^ levels can induce deleterious effects (Brini et al., 2014). Meijer et al. (2014) reported that OPs, including CPF and parathion, inhibit depolarization-evoked intracellular Ca^2+^ concentrations via voltage-gated calcium channels (Meijer et al., 2014). These effects occurred in PC12 cells following exposures to CPF and parathion at levels that did not significantly inhibit purified AChE. CPO, which is a much more efficient AChE inhibitor, also significantly decreased depolarization-evoked intracellular Ca^2+^ concentrations. Effects on Ca^2+^ signaling were only partially reversible after 20 min. Furthermore, Meijer et al. (2015) demonstrated that longer exposures (24 h) and repeated exposures (2 exposures in 24 h) inhibited voltage-gated calcium channels and depolarization-evoked intracellular Ca^2+^ concentrations (Meijer et al., 2015).

Further evidence of cell signaling deficits associated with prolonged, low to moderate CPF exposure has been demonstrated *in vivo*. Muller et al. (2014) observed that repeated CPF exposure alters rat brain electroencephalograph (EEG), a measurement that represents the summation of synchronized electrical currents in the brain. Following 7 days of 0.1, 1, and 10 mg/kg/d CPF exposure, which inhibits plasma ChE activity by 0, 50, and 80%, dose-dependent disturbances in EEG rhythms were detected, first decreasing the power spectra at low frequencies and then increasing the power spectra at frequencies higher than 4 Hz. These changes indicate disturbances in functional connectivity. In addition, somatosensory evoked potentials (SEPs), which assess the relay of sensory signals from the periphery to the brain, were altered following this same exposure paradigm. All doses of CPF decreased the negative wave amplitudes, while only the highest CPF dose (10 mg/kg/d) decreased the positive wave amplitude (Muller et al., 2014).

OPs also affect the speed of synaptic transmission. For example, following 5 days of CPF exposure at 5 mg/kg/d, levels that inhibited hippocampal AChE by <10% after a single injection and by approximately 40% after 5 injections, biphasic change in hippocampal synaptic transmission were detected in slice cultures (Speed et al., 2012). Initially, synaptic transmission through the CA3-CA1 region of the hippocampus was increased. Approximately 3 months later, however, synaptic transmission in this same region was reduced by 50%. The authors did not report deficits in neuronal survival but did observe correlation between the electrophysiological deficits induced by CPF and decreases in neuronal dendritic spine density (Speed et al., 2012).

Axonal transport comprises movement of critical nutrients, proteins, and synaptic vesicles between the soma and the axon terminal, and is a critical aspect of intercellular communication in the brain. Sciatic nerves harvested from rats exposed to 2.5, 10, 18, or 25 mg/kg/d CPF for 14 days, which exhibited plasma ChE inhibition from 30 to 60% following a single injection, showed impaired anterograde and retrograde axonal transport as measured *ex vivo* by video-enhanced differential interference contrast microscopy (AVEC-DIC) (Terry et al., 2003). Deficits in axonal transport of vesicles persisted for up to 20 days following the last day of CPF exposure. In addition, MRI studies have shown that 14-day exposures to 3 and 18 mg/kg/d CPF reduced brain AChE activity by 60–80% in rats and significantly decreased axonal transport of Mn^2+^, both at the end of the exposure period and after a 30-day washout period (Hernandez et al., 2015). CPF-induced impairments in axonal transport can partially be explained by altered motility of mitochondria and changes in motor proteins like kinesin and microtubules, as well as motor-associated proteins like tubulin (Gearhart et al., 2007; Prendergast et al., 2007; Grigoryan and Lockridge, 2009; Middlemore-Risher et al., 2011). Similar impairments in motor proteins are induced by DFP exposure, which may also impair axonal transport (Gearhart et al., 2007; Gao et al., 2016).

### Neuroinflammation

Neuroinflammation and immune responses are important mechanisms of neurotoxicity implicated in OP exposure. Here, we briefly summarize the existing data regarding the ability of OPs to modulate neuroinflammation. For a full review on the effects of OPs on inflammation and immune response (see Banks and Lein, 2012; Banks, 2015). Repeated administration of CPF at levels that do not inhibit red blood cell ChE activity induces glial fibrillary acidic protein (GFAP) expression in the mouse hippocampus (Lim et al., 2011). GFAP expression is commonly associated with gliosis, an inflammatory response of the brain to neuronal injury that usually results in hypertrophy or proliferation of glial cells, including astrocytes and microglia. *In vitro* evidence further supports these findings, showing that human fetal astrocytes treated with CPF for 1 week expressed increased levels of GFAP, IL-6, and other genes associated with proinflammatory cytokine pathways (Mense et al., 2006). Malathion exposure that inhibited hippocampal AChE by 40% also increased GFAP, specifically in the hippocampus (dos Santos et al., 2016). Likewise, DDVP activates microglia following repeated exposure *in vivo*. Animals administered 2.5 mg/kg/d DDVP for 12 weeks, a dose previously shown to inhibit AChE by 10–55% (Verma et al., 2009), exhibited microglial activation preceding neuronal degeneration of cells specifically in the SN and the CS (Binukumar et al., 2011). The SN and CS are vulnerable regions in PD and have previously been identified as targets for DDVP (Binukumar et al., 2010). Activation of microglia was accompanied by increases in proinflammatory cytokines and markers of oxidative stress, further indicating a role for neuroinflammation in DDVP-induced neurotoxicity (Binukumar et al., 2011). These studies highlight the potential role of neuroinflammation in the neurotoxicology associated with OP exposure.

### Impairment of the blood brain barrier

In a healthy nervous system, the blood brain barrier (BBB) is composed of specialized endothelial cells that form tight junctions to restrict passage of large molecules and most cells (Saunders et al., 2012). Traumatic brain injury, hypoxia, and certain chemicals increase BBB permeability. For example, in an *in vitro* model of the BBB, the OP malathion and its metabolite malaoxon decreased BBB integrity, as measured by transendothelial electrical resistance, following 2-, 4-, 8-, 16-, and 24-h exposures (Balbuena et al., 2010). Malathion and malaoxon also decreased protein levels of the endothelial tight junction proteins occludin, claudin-5, ZO1, and ZO2, which are critical for maintenance of tight junctions in a healthy BBB (Balbuena et al., 2011). Although ChE inhibition levels were not reported in this study, previous work using this model showed 20–50% AChE inhibition (Balbuena et al., 2010), levels comparable to brain AChE inhibition in previous *in vivo* reports using other OPs, such as CPF (Middlemore-Risher et al., 2010), DDVP (Verma et al., 2009), and DFP (Prendergast et al., 1997). CPF also affects the integrity of the BBB. Following 24 h exposures to CPF and CPO that inhibit ChE by 40–100%, bovine microvascular endothelial cells (BMEC) exhibited significant concentration-dependent decreases in electrical resistance (Parran et al., 2005).

## Conclusions

Despite increasing research in the field, the effects of repeated or prolonged OP exposure at low to moderate levels, both in humans and animal models, remain unclear. Evidence in humans suggests associations between occupational levels of OP exposure and deficits in executive function, neuropsychiatric issues, and neurodegenerative diseases. Effects on learning and memory in humans are repeatedly observed following occupational exposures to CPF, but less often examined with DFP and MP. Studies aimed at examining these types of exposures in animal models also produce ambiguous results. OPs, including CPF, DFP, and MP, can transiently affect learning and memory, although conflicting results exist for MP. Furthermore, malathion exposure disrupts learning and memory, although the transient nature of these effects has not been explored. Attention deficits are also often reported in occupational OP epidemiological studies. *In vivo* preclinical models demonstrate clear effects of CPF and DFP on attention and impulsivity following repeated, low to moderate exposures. These effects also appear to be transient, but are not consistently supported by PPI measurements, specifically in the case of DFP exposure. Disturbances in cytoarchitecture and neurotransmission, as well as cytotoxicity, could be responsible or contribute to executive function deficits described in both *in vivo* rodent studies and in humans, but causal studies are critically lacking.

Unless acutely toxic exposures are involved, disturbances in motility are only occasionally described in humans occupationally exposed to OPs. However, evidence from *in vivo* studies suggests that MP, DFP, DDVP, and CPF all induce transient effects on motility, as measured by open field locomotion, grip strength, and rotarod. These effects can be bidirectional, with low doses increasing activity and higher doses decreasing activity. However, these effects are not without contention, as some studies do not describe deficits in motility, specifically with CPF exposure paradigms. Disturbances in energy homeostasis and neurotransmission could account for motility deficits, but direct causality for underlying mechanisms responsible for motility issues should be explored.

In addition, behavioral correlates of affect are often examined in studies regarding OP exposure, in humans and animals alike. Epidemiological evidence for occupational OP exposures being associated with neuropsychiatric issues exists, but this body of work is problematic to interpret because affect is difficult to quantify. *In vivo* studies in preclinical models suggest that CPF, DFP, and DDVP induce depression-like behavior, as measured by FST, learned helplessness, novelty suppressed feeding, and exploratory behavior. Malathion has not been shown to alter exploratory behavior, but other behavioral paradigms related to affect have not been employed. Only one study observed changes in behavior on the EPM, describing increased risky behavior with higher levels of CPF.

**Figure 1:**
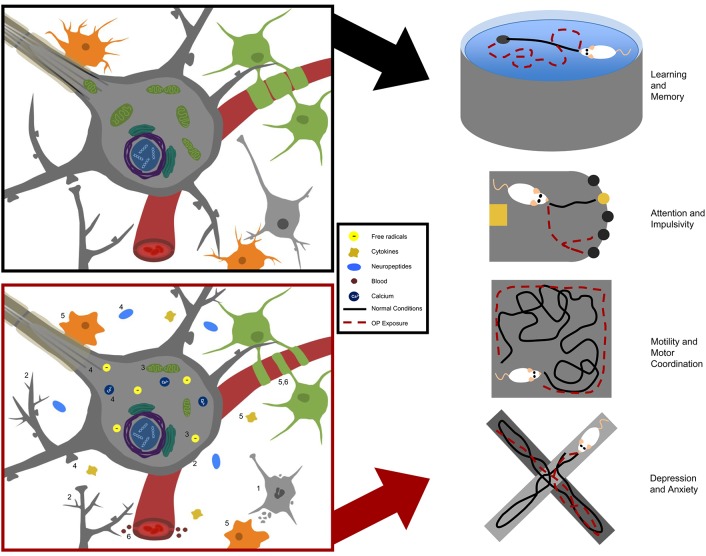
**Summary of neurotoxic effects in experimental models of occupational OP exposures**. Molecular manifestations are summarized on the left with normal physiological conditions in the black box and OP-induced effects in the red box. Numbers in the lower box correspond to numbers for specific endpoints identified in Table 6. Behavioral outcomes identified in animal models of occupational OP exposure are summarized on the right with black lines indicating normal behavior and red dashed lines indicating altered responses in OP-exposed animals.

Numerous studies have independently demonstrated the proclivity of a variety of OPs at lower, prolonged levels to induce major changes in the central nervous system, including alterations in gene expression, cell signaling pathways, and cellular ultrastructure (summarized in Table 6). However, the majority of these molecular changes have been studied *in vitro*, and more studies should be employed to confirm whether these changes also occur *in vivo*, as well as to investigate causality between these mechanisms and neurobehavioral deficits. Ultimately, each of these molecular disturbances, together or independently, could contribute to the behavioral manifestations in animal models discussed here (summarized in Table 4), including disturbances in learning and memory, attention and impulsivity, motor coordination, and depression and anxiety. These findings have been summarized in Figure 1.

## Thoughts moving forward

The studies presented in this review aid in our understanding of the effects of repeated, low- to moderate-level OP exposures.

Some important unmet needs in understanding the effects of occupational OP exposures are:

1. improvement of methods for quantifying human exposures,
2. development of more representative biomarkers of neurotoxicity,
3. deeper investigation of gene-environment interactions,
4. inclusion of more females in study design,
5. broader examination of exposures to mixtures of OPs,
6. implementation of inhalation studies, and
7. more studies to address the longitudinal effects of early-life exposures.

With respect to (1) and (2), AChE inhibition levels are currently used as a common measurement in animal studies to describe OP exposure. However, it is clear that AChE is inhibited to varying extents depending on animal or cell model, dose, duration of exposure, and specific OP. Although many of the studies presented here do not produce cholinergic crisis, despite having large reductions in ChE activity, significant but variable effects can be seen. New biomarkers of exposures and methods of identifying the extent of toxicity following these types of exposures need to be developed in order to accurately compare studies and draw valid conclusions regarding the effects of occupational OP exposures. Development of these new biomarkers would aid in improving our ability to standardize comparison of results from both *in vivo* and *in vitro* studies. In addition, application of a standardized behavioral test battery that measures an array of functional endpoints in future OP animal studies could also help facilitate comparisons between species, OPs, and varying exposure paradigms. In human studies, AChE inhibition is commonly used as a biomarker of effect, while urinary metabolite measurements are generally used as biomarkers of exposure. These measurements aid comparison of exposures across human studies, and new research is currently underway to identify and develop novel biomarkers of both exposure and effect.

With respect to (3), gene-environment interactions should be explored in regards to occupational OP exposures, as they are implicated in the etiology of nearly all diseases. PD is a great example of the importance of gene-environment interactions in disease development. For example, α-synuclein accumulation, a pathological hallmark of PD, is notoriously affected by chemicals that increase ROS (Ross and Smith, 2007). Furthermore, OPs are more strongly associated with PD in patients with certain genetic variations in paraoxonase 1 (*PON1*), aldehyde dehydrogenase (*ALDH2*), and nitric oxide synthase (*NOS1*) (Benmoyal-Segal et al., 2005; Fitzmaurice et al., 2014; Paul et al., 2016). Accordingly, it is critical to pursue research that considers the potential for gene-environment interactions in the neurotoxicity associated with OPs. Recent development of the CRISPR Cas9 system has made genetic modeling easier and quicker than ever before (Hsu et al., 2014). Implementation of this system in studying gene-environment interactions would greatly improve our understanding of how OP chemicals interact with specific genes and cellular pathways.

With respect to (4), (5), and (6), studies could more accurately and completely depict repeated low to moderate OP exposures in the workplace by including females, examining OP mixtures, and employing inhalation exposure paradigms. First, one gap in research identified through this review is understanding the effects of exposure to OP chemicals across sexes. Little is known about the effects of OPs in males vs. females (Karalliedde et al., 2003). However, certain characteristics of females may provide protection from toxicities associated with OP exposures, or alternatively render them more vulnerable to exposure. For example, females have higher paraoxonase activity (Costa et al., 2005), which is a primary OP detoxifying enzyme, and therefore may be protected from certain OP exposures. Alternatively, pregnant women pose a different concern for occupational exposure hazards, as developing fetuses are particularly at risk for adverse effects following OP exposures (Eskenazi et al., 2008). Unfortunately, as noted in Table 2, there are only two studies that met inclusion criteria for this review that investigated the effects of OPs in females, neither of which investigated behavioral endpoints. Furthermore, none of the studies compared sexes to determine whether sex differences in neurotoxic responses to OPs exist. According to the Food and Agriculture organization of the United Nations (FAO), women make up roughly 43% of the global agricultural workforce, and should therefore also be considered in these studies (FAO, 2010). In some countries, it is even common for a pregnant woman to work until the time she gives birth, and then to almost immediately return to work. While epidemiological studies have focused on the effects of these occupational exposures on children and identified correlations with ADHD, autism, and developmental disorders (Miodovnik, 2011; Burns et al., 2013), comparatively little is understood about the effects women themselves endure. It is imperative that more research be completed in females in order to begin to understand the effects of these exposures across sexes.

With specific reference to (5), both *in vivo* and *in vitro* studies should explore the effects of OP mixtures. Occupational exposures are rarely limited to a single OP, and it is possible for exposure to multiple OPs and other pesticides to have additive or synergistic effects. For example, toxicity associated with exposure to malathion was exacerbated when occurring concomitantly with carboxylesterases (CaEs) inhibitor exposure (Casida and Sanderson, 1961; Cohen and Murphy, 1971a,b; Verschoyle et al., 1982). Also, del-Rahman et al. (2004) reported greater-than additive effects of malathion when mixed with additional pesticides. In this review, only one study examined the effects of chemical mixtures, but it included an OP and organochlorine mixture, not multiple OPs.

With specific reference to (6), the purpose of this review is to examine the models used to study occupational exposures to OPs and the results pertaining to the effects of these exposures, specifically on the nervous system. We carefully selected studies that we believe best replicate human occupational exposures to OPs. In doing so, we restricted our discussion to models of prolonged dermal exposure at sub-cholinergic crisis levels to study the effects of these chemicals on neurologic endpoints. We believe exposure paradigms using topical application or subcutaneous injections best replicate the exposure parameters generally experienced by humans who are occupationally exposed to OPs. These exposure methods ensure a prolonged, systemic exposure that represents dermal exposure, the primary route in occupational settings. Another important exposure route in the occupational setting, however, is inhalation, specifically in reference to T_*O*_CP exposures in airplane-associated occupations (de Ree et al., 2014). More research into the effects of inhalation exposure is warranted due to increasing awareness for an aerotoxic syndrome among airline crewmembers, a syndrome that produces headaches, dizziness, confusion, muscle weakness, and neurobehavioral problems (de Ree et al., 2014). Unfortunately, few studies have investigated the effects of inhalation exposure to OPs on the nervous system. Therefore, we could not include them in this review. Recent improvements in inhalation and aerosolization equipment provide scientists with the opportunity to answer questions about the effects of inhaling OPs. There is a need for future studies directed toward understanding both the *in vivo* and *in vitro* effects of inhalation/aerosolization of OPs on the nervous system.

Finally, with respect to (7), the long-term effects of occupational OP exposures have not been heavily investigated in humans, but lasting neurological impairment has been reported (Jamal, 1997; Rohlman et al., 2016). Moreover, very few studies have examined the lasting effects of repeated, low- or moderate-level exposures in experimental models. *In vivo* studies examining long-term effects of occupational OP exposures included in this analysis demonstrated the transient nature of deficits induced by OPs on neurobehavior. Interestingly, however, hippocampal electrophysiology, dendritic outgrowth, and axonal transport of Mn^2+^ were altered months after exposure to CPF, but this does not correlate with the transient behavioral deficits observed *in vivo*. The potential for these molecular changes to cause long-term effects certainly warrants further investigation.

Since occupational exposures to OPs are common, and workers represent the most at-risk population for OP-associated side effects, research focused on the adverse health effects is crucial. Although the evidence linking occupational exposure to neurotoxic outcomes is contradictory, animal studies that aim to simulate exposure durations and levels consistent with occupational exposures indicate that these OP exposures induce neurologic sequelae. Furthermore, studies *in vitro* are starting to illuminate potential mechanisms by which OPs cause neurobehavioral deficits, which may inform animal and human studies. Ultimately, more research pertaining to occupational OP exposures is necessary to determine safe exposure levels, refine protection methods, establish treatment strategies, and improve workplace conditions globally.

## Author contributions

JV, DR, PL, and AP co-wrote the manuscript.

### Conflict of interest statement

The authors declare that the research was conducted in the absence of any commercial or financial relationships that could be construed as a potential conflict of interest. The reviewer JW and handling Editor declared their shared affiliation, and the handling Editor states that the process nevertheless met the standards of a fair and objective review.
